# Supplementary material for: Plasticity associated with adoption of social roles in clown anemonefish
Source: eLife. 2026 Oct 28;15:RP109871. doi: 10.7554/eLife.109871 (PMC13716389; doi:10.7554/eLife.109871)
Supplement: Peer review history

# Editor report: eLife Assessment

**DOI:** 10.7554/eLife.109871.3.sa0

**Authors:** Sonia Q Sen

**Affiliations:** Tata Institute for Genetics and Society India

**Keywords:** Solid, Valuable

Using the clownfish model, this study examines how growth, feeding, and agonistic behavior result in socially dominant or subordinate states in size- and age-matched individuals of the clownfish, *Amphiprion percula*. The authors complement this work with whole-body transcriptomics and find significant variation in genes and gene co-expression modules related to growth and satiety-related pathways, as well as ossification-related genes. They provide **solid** evidence that emerging dominants grow more, eat more, and behave more aggressively than subordinate or solitary individuals; these phenotypic differences are accompanied by distinct gene expression profiles, including variation in growth- and satiety-related pathways. The work is **valuable** in advancing our understanding of how the social environment regulates phenotypic change; however, claims regarding the mechanistic role of gene expression are only partially supported by the current analyses.

# Referee report: Reviewer #1 (Public review)

**DOI:** 10.7554/eLife.109871.3.sa1

Summary:

Overall, this is an interesting and well-written manuscript on a fascinating question in a "charismatic" model system.

Strengths:

(1) The Introduction is concise, though it might be helpful to the non-specialist reader to learn a bit more about what is known about the social control of somatic growth across diverse species (including humans), which would help to make this work more generally interesting.

(2) The experiment is well designed.

(3) The data collected are comprehensive.

(4) The complementary analysis of both feeding and aggression/submission data with and without known social roles is a neat idea and compelling!

Weaknesses:

The authors have addressed my concerns quite well. They now discuss the HPA/stress axis in some detail and also examined the extent to which growth, food intake, agonistic behavior, and/or gene expression patterns are coordinated across P1 vs P2 pairs. While still not ideal, they now provide a reasonable rationale for using whole bodies for the transcriptome analysis. Finally, the Discussion has been streamlined and is easy to follow.

# Referee report: Reviewer #2 (Public review)

**DOI:** 10.7554/eLife.109871.3.sa2

In this manuscript, the authors test growth, behavior, and gene expression in pairs of clownfish as they establish social dominance hierarchies, examining patterns of gene expression in these pairs after dominance has been established. The authors show solid evidence that emerging dominant clownfish show increased growth, aggression, and food consumption compared to their submissive or solitary counterparts, eventually adopting distinct gene expression profiles.

Major Comments:

(1) The Introduction is comprehensive, but it could be condensed. Likewise, the discussion could be condensed. There is considerable redundancy between the methods, the results, and the legend in Figure 1. The authors should consolidate and remove the redundancy.

(2) For Figure 3, the authors are showing PC2 and PC3; why is PC1 not shown? There is so much overlap between the three groups in PC2 vs PC3; it seems unlikely that researchers could conclusively identify any individual as belonging to a group based on the expression profile. The ovals shown do not capture all the points within each of the groups, and particularly the grey S oval seems misaligned with the datapoints shown.

(3) The authors indicate that the 15 replicates exhibiting the greatest size difference between P1 and P2 were selected for gene profiling. Does this mean that each of the P1 and P2 were pairs with each other? Have the authors tried examining the gene expression patterns in a paired manner? E.g., for the pairs that showed the greatest size differences, do they also show the greatest differences in gene expression? Do the P1s show the most extreme differences from P2s that also show the most extreme P2 differences? Perhaps lines on Figure 3A connecting datapoints from the P1 and P2 pairs would be informative.

(4) For the specific target pathways that are up- and downregulated in the different backgrounds, I recommend that the authors include boxplots (or heatmaps) showing the actual expression values for these targets. Figure 6 shows a heatmap for appetite-related genes, and it would be great to see a similar graph for the metabolism and glycolysis genes; it would also be informative to see similar graphs for hormonal and sexual maturation pathways as well.

(5) Particularly given that there is a relatively small number of genes enriched in the different rank conditions, I did not understand the need to do the WGCNA module analysis. I thought that an analysis of GO terms across the dataset would have been more meaningful than the GO term analysis shown in Figure 4, which considers only genes assigned to the "brown WGCNA module". This should be simplified or clarified.

(6) The authors say that they have identified coordinated changes in behaviors and the "underlying gene expression, leading to the emergence" of social roles. This is a little bit misleading, since the gene expression analysis occurred well after the behavioral and phenotypic differences emerged. Presumably, the hormonal and genetic shifts that actually caused the behavioral and phenotypic difference occurred during the weeks during which the experiment was underway, and earlier capture of the transcriptome would presumably reveal different patterns, and ones that would be considered more causative. The authors acknowledge this in 434-435, but it could be emphasized further.

(7) The authors have measured a number of differences between the different dominance classes of fish. All these differences were measured relative to the other classes, but in my view, the Solitary group was the closest to a baseline control. So, I'm not sure that it is fair to say that "P2 and S individuals showed consistent downregulation of these genes and pathways" (line 401). I encourage the authors to emphasize the differences in gene expression from the "perspective" of the P1 individuals compared to the baseline of P2 and S individuals. Line 474 says that "P2 fish showed significant upregulation" of a number of pathways. It should be very clear what that is compared to (compared to P1, presumably?)

(8) Along the same lines, the authors say in line 514 that subordinates and solitaries strategically downregulate their growth. I'm not convinced that this is the case: I would consider this growth trajectory to be the default and the baseline. I would interpret that under certain social conditions, a P1 dominant pattern of growth, behavior, and gene expression is allowed to emerge.

Comments on revised version:

The manuscript has been carefully revised. The authors have also responded adequately to all of my previous comments.

# Referee report: Reviewer #3 (Public review)

**DOI:** 10.7554/eLife.109871.3.sa3

Summary:

The authors tested the hypothesis that interactions among size- and age-matched rivals will lead to the emergence of social roles, accompanied by divergence in four aspects of individual phenotypes: growth, feeding behavior, fighting behaviors, and gene expression in clownfish.

Strengths:

The data of growth, feeding rate, and fighting behaviors support the authors claim.

Weaknesses:

The results obtained solely from the whole-body transcriptome are limited in supporting the authors' research question. However, the revised manuscript explicitly states this as a limitation.

# Author comment: Author response

**DOI:** 10.7554/eLife.109871.3.sa4

**Authors:** Lili F Vizer, Douglas Alvarado, Colleen Bove, Marcela Herrera, Annabel Hughes, Kian Thompson, Steven M Bogdanowicz, Vincent Laudet, Sarah W Davies, Pete Buston

**Affiliations:** Boston University Boston United States; Boston University Boston United States; Boston University Boston United States; Okinawa Institute of Science and Technology Graduate University Onna Japan; Boston University Boston United States; Boston University Boston United States; Cornell University Ithaca United States; Okinawa Institute of Science and Technology Graduate University Onna-son Japan; Boston University Boston United States; Boston University Boston United States

The following is the authors’ response to the original reviews.

**Public Reviews:**

**Reviewer #1 (Public review):**

Summary:

Overall, this is an interesting and well-written manuscript on a fascinating question in a "charismatic" model system.

Strengths:

(1) The Introduction is concise, though it might be helpful to the non-specialist reader to learn a bit more about what is known about the social control of somatic growth across diverse species (including humans), which would help to make this work more generally interesting.

(2) The experiment is well-designed.

(3) The data collected are comprehensive.

(4) The complementary analysis of both feeding and aggression/submission data with and without known social roles is a neat idea and compelling!

Thank you for the positive feedback!

Here, we investigate phenotypic plasticity associated with the adoption of social roles in the clown anemonefish, with strategic growth being just one aspect of that plasticity. Strategic growth, also known as social control of growth, is a fascinating form of adaptive phenotypic plasticity, whereby individuals modify their growth and size in response to fine-scale changes in social conditions (Buston & Clutton-Brock, 2022). In cooperative breeding systems with high reproductive skew, particularly fishes and mammals (possibly including humans), individuals have been shown to (i) increase growth/size on the acquisition of dominant status (Dengler-Crish & Catania, 2007; Johnston et al., 2021; Thorley et al., 2018; Van Schaik & Van Hooff, 1996; Walker & McCormick, 2009), (ii) increase growth/size when paired with size matched reproductive rivals (Huchard et al., 2016; Reed et al., 2019; this study), and (iii) decrease growth/size to avoid conflict (Buston, 2003; Heg et al., 2004; Wong et al., 2007). While strategic growth is fascinating and clearly occurring in this study, we show coordinated changes of multiple aspects of the phenotype as fish adopt social roles. Therefore, we deliberately framed the Introduction broadly to avoid biasing the reader toward viewing growth as the sole or main driver.

Weaknesses:

(1) I was surprised that the HPA/stress axis was not considered here at all. Wouldn't we expect that subordinates have increased stress axis activation, which in turn could inhibit their growth and aggressive behavior?

We also expected to see the HPA/stress axis activated in subordinates, which is why we carried out a targeted exploration of genes known to play a role in this axis. We did not find any genes that were significantly differentially expressed. We believe that there could be two explanations for this. First, from a methodological perspective, it could be due to our use of a whole-body RNA-seq, which may have masked this signal. Alternatively, the stress axis might play a more complex role than just acting as a simple on/off switch for reduced growth. Its activation may peak when competition over size is at its highest (during week one) or, conversely, it may peak later and help maintain reduced growth once hierarchies are firmly established (particularly after the dominant individual reaches its maximum size). To understand the role of the stress axis, future studies should observe how its activation varies over time. We acknowledge that the absence of a stress‑axis signal and its potential explanations were not clearly discussed in the original manuscript. In the revised version, we have addressed this in the Discussion at lines 564-567 and Methods at lines 795-797 and 802-805.

Discussion lines 564-567 and 577-580:

“These include appetite regulation (orexigenic and anorexigenic signaling), metabolic pathways (e.g., glycolysis, lactic fermentation, TCA cycle, fatty acid β-oxidation), growth-regulating pathways (GH/IGF, insulin/PI3K-AKT, mTOR, and Hippo), and potential molecular signatures to varying levels of social stress.”

Methods lines 795-797 and 802-805:

“To further investigate GE differences, we explored genes associated with growth (including thyroid signaling), appetite regulation, metabolism, and stress (corticoid) pathways across social positions.”

“A complete list of retrieved *A. percula* gene IDs were then filtered against the whole-body GE dataset, and pathways containing significant genes associated with social position were reported (Supplementary Table S2; appetite and metabolic genes shown).”

(2) To what extent are growth, food intake, agonistic behavior, and/or gene expression patterns coordinated across P1 vs P2 pairs? The lack of such an analysis seems like a missed opportunity.

We had a similar thought. Specifically, we were interested in testing the hypothesis that the final size ratio of pairs, which is indicative of the amount of conflict remaining, would predict gene expression. We examined gene expression within pairs to test for coordinated changes and repeated the analysis, accounting for the pair size ratio. In both cases, we found no clear or consistent pattern within pairs. We have included these analyses in the revised manuscript, Methods lines 781-7946-804 and 807-814, and Supplementary Materials lines 144-155 and 164-172 along with three new Supplementary Figures: Fig. S8, S9, S10.

Supplementary Materials lines 144-155 and 164-172:

“We have identified genes associated with growth and ossification that showed strong positive correlations with body size. For this set of genes, we tested the hypothesis that the final size ratio of pairs (P2 SL / P1 SL), which is indicative of the amount of remaining conflict (Wong et al., 2007), would predict variation observed in gene expression within social position (Fig. 5B). Visual inspection of the size ratio annotation in the heatmap of these genes (Supplementary Fig. S8), together with a PCA of paired individuals (P1 and P2) based on the same row Z-score values, was used to assess whether remaining conflict explained expression variation (PERMANOVA: p = 0.858; Supplementary Fig. S10A). These results suggest that size ratio between pairs was not associated with gene expression variation within social position.”

“We have identified genes associated with appetite regulation and metabolism, which were significantly downregulated in P1 individuals compared to P2 and S fish. As above, we tested the hypothesis that the final size ratio of pairs (P2 SL / P1 SL), which is indicative of the amount of remaining conflict (Wong et al. 2016), would predict variation observed in gene expression within social position (Fig. 6). We found no significant effect (PERMANOVA: p = 0.844; Supplementary Fig. S9 and S10B), indicating that size ratio does not explain gene expression differences within social positions.”

Methods lines 781-794 and 807-814:

“In the heatmap, samples (columns) were a priori grouped by social position (P1, P2, S) and subsequently ordered within each group by gene expression similarity, reflected by the column dendrogram.

Additionally, we tested the hypothesis that size ratio of pairs (P2 SL / P1 SL), which is indicative of the amount of conflict remaining (Wong et al., 2016), would predict variation in gene expression. This was assessed by visual inspection of the same heatmap with size ratio annotation using Complex Heatmaps (Gu et al., 2016), together with a PCA of the expression of these genes of paired individuals (P1, P2) only. We also performed a PERMANOVA analysis using the same row Z-score values (*adonis2* function in vegan package: Oksanen et al., 2017) accounting for social position and genetic background (clutch ID), using restricted permutations to account for the non-independence of P1 and P2 within pairs was carried out. Solitary individuals were excluded from this analysis because they lack size ratio data. We found that size ratio between pairs was not associated with gene expression variation within social position (see Supplementary Materials; Supplementary Fig. S8, S10A).”

“In the heatmap, samples (columns) and genes (rows) are grouped a priori by social position (P1, P2, S) and pathway (APT, TCA, GLY), respectively, with dendrograms reflecting expression similarity within each group. For the candidate gene heatmap, we repeated the PCA and PERMANOVA exploration and associated heatmap visualization for pairs only (as described above), to test whether final size ratio (P2 SL / P1 SL) predicted gene expression variation within social positions. We found that size ratio between pairs was not associated with gene expression variation within social position (see Supplementary Materials; Supplementary Fig. S9, S10B).”

(3) What was the rationale for using whole bodies for the transcriptome analysis? Given the hypotheses, the forebrain or hypothalamus and certain other organ systems (e.g.,liver, gonads, skin, etc.) would have been obvious candidate tissues here. I realize that cost is always a consideration, but maybe a focus on the fore-/midbrain could have been prioritized.

We decided to use whole-body samples for this initial transcriptomic analysis to capture a broad view of gene-expression differences while keeping sequencing costs and sample requirements manageable. We agree with the reviewer that future work should explore specific tissues sampled from individuals at multiple time points to disentangle transcriptomic differences across tissue types. In our revised manuscript we explicitly state the limitations and outline future steps in the Discussion at lines 553-564, as well as in the Methods section we now explicitly state the rationale behind whole-body RNA-seq and acknowledge the limitations of this approach in lines 688-692.

Discussion line 5536-564:

“In this study, we used whole-body transcriptomics, which revealed overarching expression patterns, however, we acknowledge that this approach limited our ability to detect finer-scale signals. Obviously, this approach cannot resolve tissue-specific gene expression changes (Lu et al., 2020; Roux et al., 2023; Yin et al., 2023) which are critical for a complete understanding social role differentiation, such as adjustments of behavior, appetite, and growth (a list we consider non-exhaustive). To disentangle gene expression signatures associated with socially induced phenotypes such as the strategic up- and downregulation of growth, future studies should include sampling points aligned with the onset of these physiological shifts. Moreover, to understand underlying pathways involved, tissue-specific transcriptomics will be essential. Sampling multiple tissues across multiple time points would disentangle nuanced regulatory processes underlying coordinated functional shifts leading to different social phenotypes of strategic growth.”

Methods lines 688-692:

“To initially explore overall gene expression patterns associated with coordinated changes during the emergence of social roles, whole-body RNA-seq was performed to keep both sequencing cost and sample requirements manageable. We acknowledge the limitations of this approach and consider this study a hypothesis-generating tool that lays the foundation for future studies.”

(4) Given the preceding point, why was a fold-change threshold used for assessing DEGs (supplementary Figure 3)? There is no biological justification to ever use a fold-change threshold, especially in bulk RNA-seq analysis. This is particularly true here, where wholebodies were used for RNA-seq analysis, which is a bit unusual. Relatively small cell populations (such as hypothalamic neurons that regulate growth or food intake) may show substantial gene expression variation across social types, yet will be masked by the masses of other cells in the whole body sample. However, gene expression may still vary significantly, albeit the fold-difference may be small. I therefore suggest a reanalysis that omits any fold-change threshold.

We thank the reviewer for this important point, and agree that an arbitrary fold‑change cutoff is inappropriate/unnecessary. It should be noted that this fold-change cutoff was only used in this single figure, and all other analyses used p-values from the entire dataset. We have removed the fold‑change threshold cutoff and corrected the Figure (previously Supplementary Figure 3) now Supplementary Fig. S5, and all corresponding text.

(5) Why is the analysis of color (hue, saturation) buried in the supplementary materials? Based on the hypotheses that motivated the study, color seems just as relevant as food intake, growth, and agonistic behavior, so even if the results are negative, they should be presented in the main paper.

We agree that color can be an important social signal, so we included color measurements in our experimental design. However, after careful consideration of the color results, we decided that our experimental timing and husbandry changes introduced multiple confounding factors, preventing us from drawing confident conclusions. Specifically, our fish were ≈1 month old at the transfer from larval to experimental tanks and had already begun to deepen their orange hue, before our experiment. (In the wild, they would settle at one to two weeks of age, prior to the deepening of the orange hue). Once individuals attain a certain hue, it seems that color development can be halted, but not reversed. The transfer also involved changes in lighting, tank background, and diet, factors known to strongly affect coloration (Maytin et. al., 2018). Our results show a uniform shift in orange hue and saturation across social groups, suggesting that these confounding factors might have dominated changes in hue.

For transparency, we report the color data in the Supplementary Materials, but we caution against drawing any strong conclusions. In the revised Supplementary Materials document, we added lines 231-236 recommending that future work should involve a targeted experiment to robustly test for the effect of the adoption of social roles on coloration or the effect of coloration on the adoption of social roles.

Supplementary Materials lines 231-236:

“Together, these considerations suggest that timing, environmental uniformity, and future reproductive potential may limit the expression or detection of socially mediated color plasticity under laboratory conditions. Future studies should carefully consider experimental design to minimize confounding factors and robustly test the effects of the adoption of social roles on coloration and the effects of coloration on the adoption of social roles.”

(6) The Discussion is sometimes difficult to follow. The authors may want to consider including a conceptual graphic that integrates the different aspects of growth and satiety regulation, etc., into a work-in-progress model of sorts, which would also facilitate clearer hypotheses for future research.

Thank you for flagging that parts of the Discussion are a bit difficult to follow. In the revised manuscript, we worked to improve readability of the Discussion. We also appreciate the suggestion of including a conceptual schematic. For this manuscript, we refrained from adding such a “work-in-progress” schematic, as we felt that our whole-body RNA-seq approach and single gene expression sampling time point substantially limited our ability to make predictions.

**Reviewer #2 (Public review):**

In this manuscript, the authors test growth, behavior, and gene expression in pairs of clownfish as they establish social dominance hierarchies, examining patterns of gene expression in these pairs after dominance has been established. The authors show solid evidence that emerging dominant clownfish show increased growth, aggression, and food consumption compared to their submissive or solitary counterparts, eventually adopting distinct gene expression profiles.

Major Comments:

(1) The Introduction is comprehensive, but it could be condensed. Likewise, the discussion could be condensed. There is considerable redundancy between the methods, the results,and the legend in Figure 1. The authors should consolidate and remove the redundancy.

Thank you for flagging that parts of the manuscript could be condensed, we will work on this as we revise the manuscript.

(2) For Figure 3, the authors are showing PC2 and PC3; why is PC1 not shown? There is so much overlap between the three groups in PC2 vs PC3; it seems unlikely that researchers could conclusively identify any individual as belonging to a group based on the expression profile. The ovals shown do not capture all the points within each of the groups, and particularly the grey S oval seems misaligned with the datapoints shown.

We understand the concern raised by the reviewer about the overlap among points in the PCA. We have explored PC1-PC3 and found that PC2 and PC3 showed the clearest, statistically significant clustering by social position, while PC1 did not capture any variation due to social position. We have explored whether other factors might be masking differences, such as genetic relatedness, tank effects, total read count per sample, and found that none of these factors explained sample clustering. Regarding the ellipses shown around the points, they were not intended to capture all points, but rather they show the estimated 95% multivariate t-distribution for that given social group. We revised the figure legend (Fig. 3) to clearly reflect this. . In addition, for transparency we have revised the Results section (lines 279-285) and Methods section (lines 754-765) to clarify that we have performed PCAs on (1) all genes, (2) top 50%, (3) top 25%, (4) top 5% most variable genes, and all three pairwise PC comparisons (PC1 and PC2, and PC1 and PC3) which we show in the added Supplementary Fig. 3S.

Results lines 279-285:

“PCAs were performed on all genes, and the top 50%, top 10%, and top 5% of the most variable genes, all of which showed consistent clustering across all pairwise PC comparisons (PC1 vs PC2; PC2 vs PC3; PC1 vs PC3; Supplementary Fig. S3). Of all the examined PCAs, PC2 and PC3 with all genes showed the clearest clustering by social position (p = 0.003), and revealed overall no significant difference in gene expression between P2 and S individuals, with P1 individuals exhibiting more distinct clustering (Fig. 3A; replicate‑annotated version of Fig. 3A in Supplementary Fig. S4; for all other PCAs see Supplementary Fig. S3).”

Methods lines 754-765:

“To test for overall GE patterns between social positions and genotypes, data were rlog-normalized and the effect of genotype and social position were compared through a Principal Component Analysis (PCA), followed by PERMANOVA analysis using Euclidean distances in the vegan package (Oksanen et al., 2017). To assess whether the strength of clustering by social position differed depending on the genes included, additional PCAs were performed on four gene subsets: (1) all genes, (2) the top 50%, (3) the top 10%, and (4) the top 5% of the most variable genes. For each subset, the first three principal components (PC1 vs PC2, PC1 vs PC3, PC2 vs PC3) were visualized to identify axes capturing variation associated with social position. To test for overall gene expression differences between social position and genotype, separate PERMANOVAs were performed for each PCA using the *adonis2* in the vegan package (Oksanen et al., 2017).”

(3) The authors indicate that the 15 replicates exhibiting the greatest size difference between P1 and P2 were selected for gene profiling. Does this mean that each of the P1and P2 were pairs with each other? Have the authors tried examining the gene expression patterns in a paired manner? E.g., for the pairs that showed the greatest size differences,do they also show the greatest differences in gene expression? Do the P1s show the most extreme differences from P2s that also show the most extreme P2 differences? Perhaps lines on Figure 3A connecting datapoints from the P1 and P2 pairs would be informative.

Yes, “15 replicates exhibiting the greatest size difference between P1 and P2 were selected for gene profiling” refers to pairs of P1 and P2, we made sure this is clearly stated in the revised Results (lines 266-269) and Methods (lines 686-688). Yes, we have explored gene expression data considering the size difference between pairs, and found that it showed no clear differences in gene expression patterns (see our response and manuscript edits above under Reviewer #1 point 2). We also added a version of the main text Fig. 3A which clearly labels replicates within the PCA as Supplementary Fig. S4.

Results lines 266-269:

“To test the prediction that whole-body gene expression patterns will vary with social roles once clear social positions have emerged (P1, P2 and S), we performed gene expression profiling on 45 individual fish (experimental groups containing paired individuals and corresponding solitaries; N = 15 samples per social position; Fig. 1D).”

Methods line 686-688:

“Fish from these 15 replicates (n=45 samples; 15 paired individuals and their corresponding solitaries) were individually homogenized to allow equal RNA extraction from all tissues.”

(4) For the specific target pathways that are up- and downregulated in the different backgrounds, I recommend that the authors include boxplots (or heatmaps) showing the actual expression values for these targets. Figure 6 shows a heatmap for appetite-related genes, and it would be great to see a similar graph for the metabolism and glycolytic genes; it would also be informative to see similar graphs for hormonal and sexual maturation pathways as well.

We have explored genes across a broad set of metabolic pathways (glycolysis, TCA cycle, lactic fermentation, PDH complex, cholesterol biosynthesis, fatty-acid synthesis, and beta-oxidation) and show all metabolic genes that showed significant differential expression between P1, P2, and S in Figure 6. Overall, very few metabolism-associated genes were significantly differentially expressed, which is why we decided to combine appetite-regulation and metabolism-associated genes into a single figure (Figure 6). In our revised version of the manuscript, we have modified Fig. 6 to clearly indicate which pathways the genes belong to and modified Methods section lines 795-797 and 802-805.

Methods lines 795-797 and 802-805:

“To further investigate GE differences, we explored genes associated with growth (including thyroid signaling), appetite regulation, metabolism, and stress (corticoid) pathways across social positions.”

“A complete list of retrieved *A. percula* gene IDs were then filtered against the whole-body GE dataset, and pathways containing significant genes associated with social position were reported (Supplementary Table S2; appetite and metabolic genes shown).”

We also examined hormonal pathways (glucocorticoid and thyroid signaling), but did not find genes in these pathways that were significantly differentially expressed. Finally, we would like to clarify that our samples consist of two-month-old juvenile individuals that are sexually immature —under ideal conditions, clown anemonefish can mature in one to two years, but they can also remain sexually immature for a decade or more (Buston 2004; Buston & García, 2007) — which is why we did not observe distinct molecular signatures of sexual maturation. We recognize that the sentence at line 520 was misleading, as we did not identify any gene expression signature that we could confidently associate with signs of sexual maturation. We have revised the sentence in the Discussion (used to be line 520, now line 543-544) as well as in the Methods section we added lines 601-606.

Discussion line 543-544:

“In our current study, individuals within pairs were ultimately progressing towards rank 1 dominant female and rank 2 subordinate male roles.”

Methods lines 601-606:

“All fish used in this experiment were sexually immature juveniles (one-month-old at the beginning, two-month-old at the end), as clown anemonefish reach sexual maturity between the ages of one and two years old. Also, under ideal conditions, individuals can remain sexually immature for a decade or more (Buston 2004b; Buston & García, 2007). Therefore, in this study, the emergence of social roles and associated phenotypes do not reflect changes associated with sexual maturation.”

(5) Particularly given that there is a relatively small number of genes enriched in the different rank conditions, I did not understand the need to do the WGCNA module analysis. I thought that an analysis of GO terms across the dataset would have been more meaningful than the GO term analysis shown in Figure 4, which considers only genes assigned to the "brown WGCNA module". This should be simplified or clarified.

To clarify, GO enrichment analysis does not establish correlations with traits, it only describes which functions or pathways are over-represented in a given gene set. That is why we began by using WGCNA to define gene sets (modules) that are correlated to phenotypes. Our primary rationale for WGCNA was to identify modules of co-expressed genes that show significant statistical correlation with the phenotypes of interest (social role: P1, P2, S; growth; and food intake). Pairwise differential expression analysis (Figure 3B) identified a few hundred significantly differentially expressed genes, but those tests treat genes independently and are not able to help us link coordinated changes of co-expressed genes to phenotypes of interest. Because WGCNA is blind to traits, it first identifies groups of co-expressed genes, which can help resolve gene expression patterns.

We therefore ran WGCNA on the rlog-transformed dataset to identify modules of co-expressed genes that show significant correlation with phenotypes of interest. For every module that showed such a correlation, we performed GO enrichment and carefully evaluated the resulting GO enrichment trees (see Supplementary Figs. S6, S7). The brown module was highlighted in the main text because it was one of the modules with a significant correlation to growth, and its associated GO enrichment showed clear growth-related signals that were not identified in the pairwise differential expression analysis results.

In our revised manuscript we have clarified the rationale for the analytical approaches in the Results section at lines 297-303 and Methods section lines 767-772 and 775-779.

Results lines 297-303:

“...a weighted gene co-expression network analysis (WGCNA; Langfelder & Horvath, 2008) was conducted on the rlog-transformed gene expression (GE) dataset. Unlike pairwise DEG analysis, which treats genes independently, WGCNA identifies groups of co-expressed genes (modules) and tests whether modules of eigengene expression are correlated with variation in phenotypes of interest. For modules showing significant correlations with phenotypes, gene ontology (GO) analyses were performed using Fisher`s exact tests to identify over-represented pathways within each module.”

Methods lines 767-772 and 775-779:

“To test whether gene expression patterns are correlated with observed phenotypic changes in growth and appetite across social positions, a Weighted Gene Correlation Network Analysis (WGCNA) was conducted on the rlog-transformed GE dataset (Langfelder & Horvath, 2008). WGCNA identifies groups of co-expressed genes (modules) and correlates eigengene expression of each module with phenotypes of interest to determine modules of genes whose expression correlates with trait variation.”

“For modules whose eigengene expression correlated with phenotypes of interest, gene ontology (GO) enrichment analysis was subsequently performed using Fisher's exact tests (presence/absence in a module) to identify over-represented pathways within each module across GO divisions of Biological Processes (BP), Molecular Functions (MF), and Cellular Components (CC) (Wright et al., 2015).”

(6) The authors say that they have identified coordinated changes in behaviors and the"underlying gene expression, leading to the emergence" of social roles. This is a little bit misleading, since the gene expression analysis occurred well after the behavioral and phenotypic differences emerged. Presumably, the hormonal and genetic shifts that actually caused the behavioral and phenotypic difference occurred during the weeks during which the experiment was underway, and earlier capture of the transcriptome would presumably reveal different patterns, and ones that would be considered more causative.The authors acknowledge this in 434-435, but it could be emphasized further.

We appreciate the reviewer raising this point. In the updated version of the manuscript, we have revised wording to convey that food intake, agonistic behavior, size and growth, and gene expression are all changing continuously, in response to each other and in response to social feedback (Introduction lines 133-136). An underappreciated aspect of this system (and likely many other systems) is that phenotype (including transcriptome) influences the outcome of social interactions, and the outcome of social interactions influences the phenotype (including the transcriptome). Earlier capture of the transcriptome would reveal different levels of gene expression, reflecting the state of the system at that moment in time.

Introduction lines 134-137:

“Underlying this cascade is an underappreciated aspect of this system (and likely many other systems) where phenotype (including gene expression) influences the outcome of social interactions, and the outcome of social interactions influences the phenotype (including gene expression).”

(7) The authors have measured a number of differences between the different dominance classes of fish. All these differences were measured relative to the other classes, but in my view, the Solitary group was the closest to a baseline control. So I'm not sure that it is fair to say that "P2 and S individuals showed consistent downregulation of these genes and pathways" (line 401). I encourage the authors to emphasize the differences in gene expression from the "perspective" of the P1 individuals compared to the baseline of P2and S individuals. Line 474 says that "P2 fish showed significant upregulation" of a number of pathways. It should be very clear what that is compared to (compared to P1, presumably?)

We agree with the reviewer that solitary individuals are the most intuitive baseline. Indeed, the experimental design included solitary fish because we expected they would serve as a useful control. Without social restraint, we anticipated they would show unrestricted growth, feeding, behavior, and associated gene‑expression patterns, similar to dominants.

We initially ran analyses using solitaries as the baseline, but after examining the results, which showed subordinate‑like characteristics for the solitary individuals, we concluded that solitary individuals are not an ecologically appropriate control for this context. Removing juveniles from a social context and housing them in isolation may be stressful and can affect physiology and behavior in ways that do not reflect a natural baseline. From a life‑history standpoint, solitary living is not the typical state for *A. percula*.

For these reasons, we reanalysed the dataset using the dominant (P1) as the reference to enable more ecologically meaningful comparisons (this choice was somewhat arbitrary, subordinates could also have been used as the reference). Given that gene expression is relative, we interpret results from both the dominant (P1) and subordinate (P2) perspectives in the Discussion to provide a complete view. We have clarified wording throughout the manuscript to make it clear that everything is relative (Introduction lines: 159-162 and 167-169; Methods lines: 622-625 and 726-728) as well as revised language throughout to make sure comparisons are clear.

Introduction lines 159-162 and 167-169:

“This experimental design allowed individuals equal opportunity to attempt taking on the dominant social role, however, through continuous social interactions (or in the case of solitaries, due to lack of social interactions), individuals took on varying social roles as dominant and subordinate members.”

“Given that all phenotypes are entirely dependent on social context with no intrinsic baseline in this species, we arbitrarily chose P1 (dominant) as the statistical reference group for all downstream analyses.”

Methods lines 622-625 and 726-728:

“This experimental design allowed individuals equal opportunity to attempt taking on the dominant social role, however, through continuous social interactions (or in the case of solitaries, due to the lack of it), individuals took on varying social roles as dominant and subordinate members.”

“Given that all phenotypes are entirely social context-dependent with no intrinsic baseline, in this species, we arbitrarily chose P1 (dominant) as the statistical reference group for all downstream analyses.”

(8) Along the same lines, the authors say in line 514 that subordinates and solitaries strategically downregulate their growth. I'm not convinced that this is the case: I would consider this growth trajectory to be the default and the baseline. I would interpret that under certain social conditions, a P1 dominant pattern of growth, behavior, and gene expression is allowed to emerge.

We respectfully disagree with the idea that a single baseline/reference growth trajectory exists for any individual of this species. Growth of individuals is entirely social context-dependent: neither fast nor slow growth represents an inherent baseline. When two size‑matched juveniles meet and compete to establish dominance, accelerated growth is the expected trajectory. By contrast, juveniles joining an existing hierarchy are expected to exhibit reduced growth, which minimizes conflict and facilitates their social integration. Unlike species that show non socially mediated growth trajectories, clown anemonefish do not have a context‑independent growth rate, rather, individuals constantly readjust their growth according to their immediate social environment (Buston 2003).

Therefore, growth trajectories must be considered from the perspective of all group members, because they emerge from interactions among individuals rather than reflecting an intrinsic baseline. In this study, we were interested in the establishment of dominance hierarchy and how individuals adjust their phenotypes during this process. By experimentally pairing size‑matched rivals, both individuals are initially expected to pursue the dominant trajectory, and thus neither individual represents a default state. Instead, the outcome reflects a social decision, after which both individuals reinforce their emerging social roles through coordinated changes. See our response and manuscript edits above under Reviewer #2, point 7 (public reviews).

**Reviewer #3 (Public review):**

Summary:

The authors tested the hypothesis that interactions among size- and age-matched rivals will lead to the emergence of social roles, accompanied by divergence in four aspects of individual phenotypes: growth, feeding behavior, fighting behaviors, and gene expression in clownfish.

Strengths:

The data on growth, feeding rate, and fighting behaviors support the authors' claims.

Thank you for the positive feedback!

Weaknesses:

Gene analysis conducted in this study is not sufficient to clarify how the relevant genes actually regulate growth and behavior.

The information obtained from whole-body gene expression analysis is very limited.Various gene expression is associated with the regulation of fighting behaviors, food intake, growth, and metabolism, and these genes are regulated differently across tissues, even within a single individual. Gene expression analysis should be performed separately for each tissue.

We understand the reviewer’s concern about whole‑body transcriptomes and agree that tissue‑specific sampling would provide greater resolution of the mechanisms linking gene expression to growth, agonistic behaviors, and food intake. For this initial study, however, we deliberately chose whole‑body samples to capture a broad, unbiased view of gene expression differences while keeping sequencing costs and sample requirements manageable. We explicitly acknowledge the resulting interpretational limits in the Discussion (lines 497; 553–5647), and suggest in the last paragraph that the patterns reported here should be used to build on in future studies exploring targeted, tissue‑specific hypotheses. See our response and manuscript edits above under Reviewer #1, point 3 (public reviews).

Clownfish undergo sex change depending on social status and body size, as the authors mention in the manuscript. Numerous gene expressions are affected by sex change. It is unclear how this issue was addressed.

We thank the reviewer for raising this point. Sex change and sexual maturation can indeed drive major transcriptional shifts in clown anemonefish, but our experiment did not encompass such a life‑history transition. All individuals in this experiment were juveniles (≈1 month old at the start, ≈2 months old at the end) and were sexually immature at these ages. Clown anemonefish reach sexual maturation around one to two years under ideal conditions, can delay sexual maturation for years under normal conditions (Buston 2004; Buston & García, 2007), and sex change in the genus Amphiprion is known to take over ~5 months (Moyer & Nakazono, 1978). Accordingly, individuals in this study were not sexually mature, and sex change was not biologically plausible over the five-week experimental period of our study. We recognize that the sentence at line 520 may be misleading, as we did not identify any gene expression signature that we could confidently associate with signs of sexual maturation. During the revisions we made sure that it is clearly stated that the fish in this study were sexually immature. See our response and manuscript edits above under Reviewer #2, point 4 (public reviews).

**Recommendations for the authors:**

**Reviewing Editor Comments:**

While appreciating the work presented in the manuscript, we note a few common concerns that the authors could prioritise in their revisions:

(1) The transcriptomics

The authors have used whole-body RNA-seq and so are constrained in the kind of mechanistic or tissue-specific insight it can really provide. There are also questions about how far the authors can go in linking these data to causation, given that sampling happened after the phenotypes had already diverged.

We therefore recommend tempering the causal language, clarifying the rationale for their analytical choices (WGCNA, fold-change threshold...), and more explicitly discussing the limitations of the whole-body RNA-seq and what can (and cannot) be concluded from it.

We thank the editors for these recommendations. We have addressed each point as follows:

Tempering causal language: We have revised our manuscript and tempered with language throughout to remove wording of “influence” or “cause” and instead used “associated with”, “correlated with”, or “suggesting”, as appropriate. We made changes to our Abstract (lines 39-41), Introduction (lines 115-118, 121-123), and Discussion (lines 431-434, 571-574), and Methods sections (lines 795-797). Whereas other parts of the manuscript already used non-causal language such as Discussion lines 333, 355, 358, 381, 437, etc.

Abstract lines 39-41:

“Here we identify associations between changes in gene expression, growth, and feeding behavior regulation that reinforce social role differentiation during dominance hierarchy formation in clownfish.”

Introduction lines 115-118, 121-123:

“Gene expression profiling offers a powerful approach to uncover coordinated changes in gene expression across multiple pathways, providing insight into associations between the development of social role-specific phenotypes and their underlying proximate mechanisms.”

“Its well-annotated genome (Lehmann et al., 2019) enables transcriptomic analyses that can help characterize the molecular patterns associated with these processes.”

Discussion lines 431-434, 571-574:

“To explore underlying GE differences, we examined genes known to be associated with appetite regulation and metabolism in *A. ocellaris* (Herrera et al., 2025), and found downregulation of these genes in P1 individuals compared to P2 and S.”

“Together, these approaches provide a powerful framework for uncovering the dynamic, context-dependent mechanisms that regulate strategic growth and coordinated changes associated with the establishment and maintenance of dominance hierarchies in social vertebrates.”

Methods lines 795-797:

“To further investigate GE differences, we explored genes associated with growth (including thyroid signaling), appetite regulation, metabolism, and stress (corticoid) pathways across social positions.”

WGCNA rationale: We have revised our Results and Methods sections to explicitly define why we used WGCNA and GO enrichment analysis, and how these analyses provided more information than simple pairwise differential gene expression analysis. See our response and manuscript edits above under Reviewer #2, point 5, (public reviews).

Fold-change threshold: In our revised manuscript, we have removed the arbitrary imposed fold-change threshold cutoff, which was only used in that single figure. See our response and manuscript edits above under to Reviewer #1, point 4, (public reviews).

Whole-body RNA-seq limitations: We have revised the Methods and Discussion sections, and see our response and manuscript edits above under Reviewer #1, point 3 (public reviews).

(2) Framing and interpretation

Several of the reviewers' comments flag that the manuscript overstates coordination or "strategic" regulation, or where what's being treated as the baseline/derived isn't clear (for example, whether P2/S are actively downregulating or whether P1 represents the divergent trajectory). We recommend revisiting any wording that implies stronger mechanistic inferences than the data actually support (and defining more clearly what is meant by baseline and socially induced state).

We thank the editors for these comments and feedback. We have revised the manuscript to clearly state that our system does not have the traditional baseline/socially induced states, rather it is all social context-dependent. In particular, we have adjusted the wording throughout the manuscript to make it clear that everything is relative, and we clarified that our choice of P1 as the reference group was arbitrary. We have made revisions in the Introduction (lines 159-162 and 167-169) and Methods (lines 622-625 and 726-728). See our response and manuscript edits above under Reviewer #2, points 7 and 8, (public reviews).

We have also revised the manuscript to temper with wording that could be interpreted as implying stronger mechanistic or directional conclusions than our data allow. We adjusted language throughout to remove wording of “influence” or “cause” and instead used “associated with”, “correlated with”, or “suggesting”, as appropriate. See our response and manuscript edits above under Reviewing Editor Comments, (1) the transcriptomics (recommendations to authors).

**Reviewer #1 (Recommendations for the authors):**

(1) The reader would benefit from a brief overview of the physiology and molecular basis of somatic growth, regulation of food intake, and aggressive behavior, especially in teleost fishes. This would also help the authors with formulating hypotheses that are a bit more explicit when it comes to the transcriptomic part of the study.

We thank the reviewer for this suggestion, however, as another reviewer raised concerns about the length of the Introduction as is and we felt that adding detailed paragraphs on the physiology and molecular basis of somatic growth, food intake regulation, and aggressive behavior would significantly increase the length of our Introduction.

As a compromise, we have revised the relevant sections of the Introduction (lines 109-115) to point readers to key review papers and primary literature where the molecular basis of these traits is covered in detail. We believe this approach balances the need for mechanistic context with manuscript length constraints, while also allowing readers with specific interests to follow up with the relevant literature.

Introduction lines 109-115:

“Most studies, often conducted without relevant social context of an individual or in species entirely lacking dominance hierarchies, have examined single pathways to uncover variation in coloration (Salis et al., 2019, 2021), appetite regulation (see review for teleosts: Volkoff, 2019), behavior (Bender et al., 2006; Renn et al., 2008; Santema et al., 2013; Solomon-Lane et al., 2022; see review for teleosts: St-Cyr & Aubin-Horth, 2009), and growth (Beckman, 2011; Lu et al., 2020; see reviews for teleosts: Reinecke, 2010; Zhou et al., 2024), leaving the broader molecular shifts associated with social role adoption unresolved.”

(2) I certainly agree with that statement that "Understanding the proximate mechanisms that facilitate phenotypic adjustments is key to disentangling whether phenotypes are the cause or consequence of social rank" (line 90f.), though maybe the authors can elaborate a bit, as this relationship is quite dynamic and obviously goes both ways.

We agree that this relationship is dynamic and bidirectional, and we have revised the Introduction to reflect this more explicitly. In particular, we added text noting that, in social vertebrates, phenotype, including gene expression, can both influence and be influenced by social interactions, often through continuous feedback loops rather than a clear directional relationship (Introduction lines 93-96). We also revised the experimental framing in the Introduction and Methods (Introduction lines 159-162 and 167-16972; Methods lines 622-625 and 726-728) to emphasize that phenotypes are socially context-dependent in *A. percula* and that the statistical reference group was chosen for analytical clarity rather than as a true biological baseline.

Introduction lines 93-96, 159-162 and 167-169:

“This is further complicated by the fact that, in social vertebrates, phenotype (including gene expression) can both influence and be influenced by social interactions, often through continuous feedback loops rather than a clear directional relationship.”

“This experimental design allowed individuals equal opportunity to attempt taking on the dominant social role, however, through continuous social interactions (or in the case of solitaries, due to lack of social interactions), individuals took on varying social roles as dominant and subordinate members.

“Given that all phenotypes are entirely dependent on social context with no intrinsic baseline in this species, we arbitrarily chose P1 (dominant) as the statistical reference group for all downstream analyses. Given that all phenotypes are entirely social context-dependent with no intrinsic baseline in this species, we arbitrarily chose P1 (dominant) as the statistical reference group for all downstream analysis.”

Methods lines 622-625 and 726-728:

“This experimental design allowed individuals equal opportunity to attempt taking on the dominant social role, however, through continuous social interactions (or in the case of solitaries, due to the lack of it), individuals took on varying social roles as dominant and subordinate members. This experimental design allowed individuals equal opportunity to attempt taking on the dominant social role; however, through continuous social interactions (or in the case of solitaries, due to the lack of it), individuals took on varying social roles as dominant and subordinate members.”

“Given that all phenotypes are entirely social context-dependent with no intrinsic baseline, in this species, we arbitrarily chose P1 (dominant) as the statistical reference group for all downstream analyses. Given that all phenotypes are entirely social context-dependent with no intrinsic baseline in this species, we arbitrarily chose P1 (dominant) as the statistical reference group for all downstream analysis.”

(3) Much of the information in the last paragraph of the Introduction (lines 151ff.) is best presented in the Methods section.

We respectfully disagree with this suggestion. The eLife journal format does not include a standalone Methods section preceding the Results, so we expect most readers not to consult the Methods before reading the Results. We believe it is important to briefly orient the reader to the experimental approach and the ideas tested, thus providing some context before they encounter the Results and Discussion.

(4) What count (TPM or similar) and abundance (above count threshold in fraction of samples) thresholds were used for the transcriptome analysis? Maybe I missed it, but how many genes were in the analysis?

We thank the reviewer for pointing this out. We have updated our Methods section (lines 714-719) to explicitly include filtering steps used as well as included the total number of genes that were used in downstream analysis.

Methods lines 714-719:

“The read count file was then imported into R version 4.3.1 (R Core Team, 2021), size factors were estimated for each sample using the median ratio method in DESeq2 (Love et al., 2014) to account for differences in sequencing depth across samples. No outlier samples were identified, and all samples (n=45) were retained for downstream analyses. Genes with a mean raw count <10 were then removed, retaining 24,840 genes for downstream analyses.”

(5) Were all these genes used in PCA, and if so, why? Would it not make more sense to only use the 50% or 25% most variable genes (which would likely enhance the separation of social types)? Also, did the authors inspect higher-order PCs to see whether any of them separate the social types or separate samples according to some other variable(e.g., size, hue, feeding, any technical factors, etc.)?

We explored PCAs using four gene subsets: all genes, the top 50%, top 10%, and top 5% most variable genes, and all pairwise PC comparisons (PC1 vs PC2, PC1 vs PC3, PC2 vs PC3; Supplementary Fig. S3). Of all these examined PCAs, PC2 and PC3, with all genes, showed the clearest, statistically significant clustering by social position, and more stringent filtering did not strengthen this signal. We therefore retained all genes in the primary analysis to avoid imposing arbitrary filtering thresholds that could exclude biologically relevant low-variance genes. See our response and manuscript edits above under Reviewer #2, point 2, (public reviews).

Regarding the inspection of higher-order PCs for potential confounding variables, we examined whether genetic background (clutch ID), tank identity, and sequencing depth explained clustering patterns across PC1–PC3 and found no evidence that any of these variables (PCA for sequencing depth not shown). See our response and manuscript edits above under Reviewer #2, point 2, (public reviews).

We did not explicitly explore whether body size at week 5 drove any of the observed separation among social positions. Rather, we investigated whether size ratio, an indicator of the amount of remaining conflict within a pair, could drive gene expression variation within social positions. See our response and manuscript edits above under Reviewer #1, point 2, (public review).

Regarding orange hue, we do not believe that orange hue at week 5 (the time point at which whole-body samples were collected for gene expression profiling) represents a meaningful socially mediated result. See our response and manuscript edits above under Reviewer #1, point 5, (public reviews).

Considering food intake, we chose not to explore this as a potential driver of PC variation because food intake could not be reliably assigned to all individuals at week 5. For a subset of pairs, individuals could not be confidently distinguished in videos, and we did not want to make assumptions that could introduce biases into the analysis.

(6) Figures 5/6: Based on the gene expression shown in the heatmaps, I cannot see how the samples would cluster so cleanly by social type. Consider a bootstrapping analysis and provide bootstrap values and "confident" nodes. Also, what does "matrix" in the legend refer to? I assume some measure of gene expression level, maybe z-scored?

We thank the reviewer for flagging this point. We acknowledge that the samples do not cluster freely by social position in the heatmaps and we have revised our Methods (see our response and manuscript edits above under Reviewer #1, point 2, public reviews) and Figure captions to clearly reflect this. To clarify, samples in Figures 5 and 6 were not ordered by unsupervised hierarchical clustering; rather, they were first grouped a priori by social position and then ordered within each group by similarity. We chose this presentation because it best illustrates the gene expression patterns associated with social position, which is the primary focus of the manuscript. We have updated the figure legends of Figures 5 and 6, to clarify that the color scale previously denoted as matrix in the heatmap represents row-scaled Z-scores of normalized gene expression values.

To assess whether social position explains a gene expression variation in an unsupervised framework, we performed a PCA followed by PERMANOVA (using the *adonis2* function in R, 999 permutations, Euclidean distance; see Author response image 1). Both analyses used the same row Z-score-scaled expression values as shown in Figures 5 and 6. Results showed a significant effect of social position on gene expression (PERMANOVA: A: social position p <0.001, B: social position p < 0.001; marginal clutch ID significance p=0.015), which was primarily driven by dominant individuals (P1) being significantly different from both subordinate (P2) and solitary (S) individuals. To include these into our manuscript.

**Author response image 1.** Principal component analysis (PCA) of gene expression based on heatmaps of (A) growth, (B) appetite, and metabolism genes. PCA was performed on gene sets of the main text (growth: Fig 5B and appetite and metabolism: Fig. 6), using row Z-score scaled expression values, consistent with the heatmap scaling. Each point represents one individual. Ellipses represent 95% confidence intervals around each social position group. PERMANOVA results (*adonis2*; shown in the bottom right corners).

(7) I applaud the authors for considering genetic/relatedness effects in their experimental design and analysis, but I am confused by the microsatellite vs. SNP analyses: why even use microsatellites in this day and age? Were only the SNP data used as the source of genetic information? This should be clarified.

In our experiment, paired individuals were initially assigned a temporary rank based on their size at the start of the experiment; however, the initially larger individual (often only by a tenth of a mm) does not necessarily emerge as the dominant (P1). To avoid any assumptions regarding the identity of individuals in given social positions at the end of the experiment, we needed to verify that individuals within pairs were correctly identified. For the 45 individuals included in the gene expression dataset, this was done by calling SNPs directly from the TagSeq data. For the remaining 36 individuals not included in the gene expression dataset, we opted for microsatellite genotyping, as a validated panel with established markers was already available from a previous study (Rueger et al., 2025), making it a reliable and cost-effective solution. We have clarified the text in the Methods (lines 630-636) and Supplementary Materials (lines 42-49 and 65-67).

Methods lines 630-636:

“This step was necessary to avoid assumptions regarding social roles and fish identity. At the beginning of the experiment, individuals within pairs were provisionally assigned a social rank based on initial body size; as size differences were negligible (often less than 0.1mm), the initially larger individual does not necessarily emerge as the dominant (P1). To correct this assumption, identities were verified at the end of the experiment using either microsatellite genotyping or SNPs called from TagSeq data, depending on whether individuals were included in the gene expression dataset (see Supplementary Materials).”

Supplementary Materials lines 42-49 and 65-67:

“This step was necessary to avoid assumptions regarding social roles and fish identity, and the microsatellite method allowed for a reliable and cost-effective solution as a panel with established markers was already available from a previous study (Rueger et al., 2025). At the beginning of the experiment, individuals within pairs were provisionally assigned a social rank based on initial body size; as size differences were negligible (often less than 0.1 mm), the initially larger individual does not necessarily emerge as the dominant (P1). To correct this assumption, identities were verified at the end of the experiment.”

“Similarly, for the remaining 45 individuals, we applied a necessary correction step to avoid assumptions regarding social role and fish identity. For these 45 individuals, we called SNPs from our TagSeq data to assign clutch identity.”

**Reviewer #2 (Recommendations for the authors):**

(1) Line 520 indicates that individuals showed early gene expression signatures of sexual maturation, but I did not see where those results were presented.

We have addressed this recommendation, the claim “individuals showed early gene expression signatures of sexual maturation” was incorrect and has been removed from the revised manuscript (Discussion lines 543-544). We have also updated the Methods section (lines 601-606) to explicitly clarify that all individuals were sexually immature juveniles throughout the experiment. See our response and manuscript edits above under Reviewer #2, point 4, (public reviews).

(2) The paragraph starting at line 409 refers to GE profiles. I was confused about what that was.

Do the authors mean GO profiles?

We did not find any modules using GO enrichment analysis which showed strong enrichment of appetite- and metabolism-related GO terms. Therefore, we looked for differentially expressed genes in the rlog-normalized gene expression dataset that are known to be associated with appetite regulation and metabolism. We have revised the Discussion (lines 431-434) and Methods (lines 812-815) to explicitly state what we are referring to and avoid confusion.

Discussion lines 431-434:

“In our study, P1 individuals showed increased food intake compared to P2 individuals. To explore underlying GE differences, we examined genes known to be associated with appetite regulation and metabolism in *A. ocellaris* (Herrera et al., 2025), and found downregulation of these genes in P1 individuals compared to P2 and S.”

Method lines 802-805:

“A complete list of retrieved *A. percula* gene IDs were then filtered against the whole-body GE dataset, and pathways containing significant genes associated with social position were reported (Supplementary Table S2; appetite and metabolic genes shown).”

(3) I saw several typos, e.g., Vulcano in Supplementary Figure 3.

Thank you for flagging this. We have addressed typos such as Supplementary Fig. S4 (used to be Supplementary Fig S3) See our response and manuscript edits above under Reviewer #1, point 4 (public reviews).

(4) The personal observations cited in 495 should be more explicit. Which author made these observations, over how long, in how many instances?

We thank the reviewer for this comment. We have updated the text to explicitly name the authors who made these observations, to clarify that they were made during two independent long-term field studies, and to note that this pattern was observed in 8 or more instances across the two field studies (Discussion lines 516-520).

Discussion lines 516-520:

“Similar patterns have been anecdotally observed in the wild, where juvenile clownfish remained small for extended periods (over four months) following the loss of a dominant partner, only initiating changes in social role towards dominant characteristics upon the arrival of a new group member (personal observations of 8+ instances during two long-term independent studies by Pete Buston and Lili Vizer).”

References:

Buston, P. (2003). Forcible eviction and prevention of recruitment in the clown anemonefish. Behavioral Ecology, 14(4), 576–582. https://doi.org/10.1093/beheco/arg036

Buston, Peter M. (2004). Territory inheritance in clownfish. Proceedings of the Royal Society B: Biological Sciences, 271(SUPPL. 4), 252–254.

Buston, P. M., & García, M. B. (2007). An extraordinary life span estimate for the clown anemonefish Amphiprion percula. Journal of Fish Biology, 70(6), 1710–1719. https://doi.org/10.1111/j.1095-8649.2007.01445.x

Buston, P., & Clutton-Brock, Tim. (2022). Strategic growth in social vertebrates (WITH REVIEWER COMMENTS). Trends in Ecology & Evolution, 37(8), 694–705. https://doi.org/10.1016/j.tree.2022.03.010

Dengler-Crish, C. M., & Catania, K. C. (2007). Phenotypic plasticity in female naked mole-rats after removal from reproductive suppression. THE JOURNAL OF EXPERIMENTAL BIOLOGY.

Heg, D, Bender, N, & Hamilton, I. (2004). Strategic growth decisions in helper cichlids. Proceedings of the Royal Society of London. Series B: Biological Sciences, 271(suppl_6). https://doi.org/10.1098/rsbl.2004.0232

Huchard, E, English, S, Bell, M B. V., Thavarajah, N, & Clutton-Brock, T. (2016). Competitive growth in a cooperative mammal. Nature, 533(7604), 532–534. https://doi.org/10.1038/nature17986

Johnston, R A., Vullioud, P, Thorley, J, Kirveslahti, H., Shen, L., Mukherjee, S., Karner, C. M., Clutton-Brock, T, & Tung, J (2021). Morphological and genomic shifts in mole-rat ‘queens’ increase fecundity but reduce skeletal integrity. eLife, 10, e65760. https://doi.org/10.7554/eLife.65760

Maytin, Alexander K., Davies, Sarah W., Smith, Gabriella E., Mullen, Sean P., & Buston, Peter M. (2018). De novo transcriptome assembly of the clown anemonefish (Amphiprion percula): A new resource to study the evolution of fish color. Frontiers in Marine Science, 5(AUG), 1–11.

Moyer, J. T., & Nakazono, A. (1978). Protandrous Hermaphroditism in Six Species of the Anemonefish Genus Amphiprion in Japan (No. 2). The Ichthyological Society of Japan. https://doi.org/10.11369/jji1950.25.101

Reed, C., Branconi, R., Majoris, J., Johnson, C., & Buston, P. (2019). Competitive growth in a social fish. Biology Letters, 15(2), 20180737. https://doi.org/10.1098/rsbl.2018.0737

Rueger, Theresa, Bhardwaj, Anjali Kristina, Turner, Emily, Barbasch, Tina Adria, Trumble, Isabela, Dent, Brianne, & Buston, Peter Michael. (2022). Vertebrate growth plasticity in response to variation in a mutualistic interaction. Scientific Reports, 12(1), 11238.

Thorley, J, Katlein, N, Goddard, K, Zöttl, M, & Clutton-Brock, T. (2018). Reproduction triggers adaptive increases in body size in female mole-rats. Proceedings of the Royal Society B: Biological Sciences, 285(1880), 20180897. https://doi.org/10.1098/rspb.2018.0897

Van Schaik, C P., & Van Hooff, J A. R. A. M. (1996). Toward an understanding of the orangutan’s social system. In Linda F. Marchant, Toshisada Nishida, & William C. McGrew (Eds.), Great Ape Societies (pp. 3–15). Cambridge University Press. https://doi.org/10.1017/CBO9780511752414.003

Walker, S P. W., & McCormick, M I. (2009). Sexual selection explains sex-specific growth plasticity and positive allometry for sexual size dimorphism in a reef fish. Proceedings of the Royal Society B: Biological Sciences, 276(1671), 3335–3343. https://doi.org/10.1098/rspb.2009.0767

Wong, M. Y. L., Buston, P. M., Munday, Philip L., & Jones, Geoffrey P. (2007). The threat of punishment enforces peaceful cooperation and stabilizes queues in a coral-reef fish. Proceedings of the Royal Society B: Biological Sciences, 274(1613), 1093–1099. https://doi.org/10.1098/rspb.2006.0284
